# Water Age Effects on the Occurrence and Concentration of *Legionella* Species in the Distribution System, Premise Plumbing, and the Cooling Towers

**DOI:** 10.3390/microorganisms10010081

**Published:** 2021-12-31

**Authors:** Alshae R. Logan-Jackson, Joan B. Rose

**Affiliations:** 1 Department of Microbiology and Molecular Genetics, Michigan State University, East Lansing, MI 48824, USA; 2 Department of Fisheries and Wildlife, Michigan State University, East Lansing, MI 48824, USA; rosejo@msu.edu

**Keywords:** *Legionella pneumophila*, *Legionella longbeachae*, *Legionella bozemanii*, *Legionella micdadei*, *Legionella anisa*, water age, building water quality

## Abstract

In this study, droplet digital PCR^TM^ (ddPCR^TM^) was used to characterize total *Legionella* spp. and five specific *Legionella* species from source (groundwater) to exposure sites (taps and cooling towers). A total of 42–10 L volume water samples were analyzed during this study: 12 from a reservoir (untreated groundwater and treated water storage tanks), 24 from two buildings (influents and taps), and six from cooling towers, all part of the same water system. The approximate water age (time in the system) for all sample locations are as follows: ~4.5, 3.4, 9.2, 20.8, and 23.2 h (h) for the groundwater to the reservoir influent, reservoir influent to the reservoir effluent, reservoir effluent to building Fa (building names are abbreviated to protect the privacy of site location), building ERC and the cooling towers, respectively. Results demonstrated that gene copies of *Legionella* spp. (23S rRNA) were significantly higher in the cooling towers and ERC building (*p* < 0.05) relative to the reservoir and building Fa (closest to reservoir). *Legionella* spp. (23S rRNA) were found in 100% (42/42) of water samples at concentrations ranging from 2.2 to 4.5 Log10 GC/100 mL. More specifically, *L. pneumophila* was found in 57% (24/42) of the water samples, followed by *L. bozemanii* 52% (22/42), *L. longbeachae* 36% (15/42), *L. micdadei* 23% (10/42) and *L. anisa* 21% (9/42) with geometric mean concentrations of 1.7, 1.7, 1.4, 1.6 and 1.7 Log_10_ GC/100 mL, respectively. Based on this study, it is hypothesized that water age in the distribution system and the premise-plumbing system as well as building management plays a major role in the increase of *Legionella* spp., (23S rRNA) and the diversity of pathogenic species found as seen in the influent, and at the taps in the ERC building—where the building water quality was most comparable to the industrial cooling towers. Other pathogenic *Legionella* species besides *L.* *pneumophila* are also likely amplifying in the system; thus, it is important to consider other disease relevant species in the whole water supply system—to subsequently control the growth of pathogenic *Legionella* in the built water environment.

## 1. Introduction

*Legionella* was first described and classified over 40 years ago [1,2]. Since its discovery, there have been 61 identified *Legionella* species [3], of which 28 have been isolated from human specimens associated with disease [3]. *Legionella pneumophila* serogroup 1 is the most well-known and studied *Legionella* species, as it is most often identified as the etiologic agent of Legionnaires’ Disease. *Legionella pneumophila* accounts for more than 90% of Legionnaires’ Disease cases [4,5] followed by *L. micdadei*, *L. bozemanii*, and *L. longbeachae* while other species such as *L. anisa* are rarely found to cause disease [6,7,8,9].

Currently, in the United States (U.S.), the incidence rate of Legionnaires’ Disease is increasing with an annual rate of 550%, corresponding to a range of 0.4 to 2.2 reported cases per 100,000 population from 2000 to 2017 [10]. *Legionella pneumophila* is the cause of most drinking water disease outbreaks in contrast to other water-related pathogens in the U.S. [11]. 

A large percentage of the U.S. population gets its drinking water from groundwater [12]. In the U.S., groundwater withdrawal for public supply accounts for approximately 39% of the total usage [12]. Even in Michigan surrounded by the Great Lakes, total groundwater usage is about 700 million gallons per day [13]. Moreover, there is an estimation of 1.7 million people in Michigan that rely on municipal water supplies utilizing groundwater as their primary drinking water source [13]. In groundwater, *Legionella* has been found in several studies to range in concentrations from 10^1^ to 10^4^ CFU/100 mL [14,15,16,17]. Groundwater sources are notorious for having iron concentrations, and this may be considered problematic [18] as iron is a micronutrient for the growth of *Legionella* [19]. 

*Legionella* bacteria are known to colonize engineered water systems such as premise plumbing, and cooling towers [20,21]. For example, *L. pneumophila* serogroup 1 occurred more than once in 7% (5/68) of cold water taps at various concentrations by a PCR method [20]. *Legionella* spp. have also occurred in 39% (78/196) of cooling towers samples collected, 27% (53/196) and 20% (40/196) of which were *L. pneumophila* and *L. pneumophila* serogroup 1, respectively [21]. In these environments, *Legionella* can be aerosolized and potentially inhaled from showers, faucets, hot tubs/swimming pools, and cooling towers [22]. However, there are only a few limited studies on specific pathogenic *Legionella* species other than *L. pneumophila* in drinking water supply systems and cooling towers. For example, there has been a characterization study of pathogenic *Legionella* species in hot water systems by MALDI-TOF and 17 *Legionella* species were identified. However, there were only two species that were mostly identified, *L. pneumophila* (identified isolates: 40,234/47,924) and *L. anisa* (identified isolates: 4307/47,924) [23]. Among the 2685 and 877 warm water installations that were positive with *L. pneumophila* and *L. anisa*, 13% (377/2685) and 30% (266/877) of the samples co-occurred with other *Legionella* species, respectively [23]. Another study detected *Legionella* species in kitchen sinks in private residences and restroom sinks in public buildings by PCR amplification and sequencing and the frequently detected species were *L. pneumophila*, *L. fairfieldensis*, and *L. dresdeniensis* [24]. Lesnik et al., 2016 [25] evaluated pathogenic *Legionella* species in a drinking water supply system by single-stranded conformation polymorphism and the most-abundant phylotypes were *L. pneumophila* and *L. longbeachae*. However, both studies [24,25] did not specifically detail the proportion of the samples that were *L. pneumophila* and non-*L. pneumophila—*the authors showed the phylogenetic relatedness of *Legionella* genus specific species. Thus, it was not possible to determine the co-occurrence of *L. pneumophila* and non-*L. pneumophila* species in individual, specific samples. Recently, Tsao et al. (2019) [26] also evaluated pathogenic *Legionella* species in cooling towers using 16 and 18S rRNA gene amplicon sequencing and out of 100% relative abundance, the frequently detected were *L. rowbothamii* (0.060%) and *L. worsleiensis* (0.030%); this study investigated the co-occurrence by the bacterial phlya but not by the species level. Llewellyn et al. [21] also detected multiple *Legionella* species in cooling towers by culture, and out of 144 *Legionella* isolates, the most common species were *L. pneumophila* (53%), *L. anisa* (22%), and *L. rubrilucens* (9%) [21]. Pereira et al. (2017) [27] evaluated pathogenic *Legionella* species in cooling towers using universal primers 16S rRNA (PCR) and genus-specific deep sequencing (next-generation sequencing) and out of 100% relative abundance, the most frequently detected species were *L. anisa* (19.2%), *L. micdadei* (18.5%), and *L. pneumophila* (18.4%). While both studies [21,27] described the proportion of the samples that were positive for *L. pneumophila* relative to non-*L. pneumophila*, neither author described the co-occurrence of *L. pneumophila* and non-*L. pneumophila* in any given sample. Logan-Jackson et al., 2021 [28] investigated several pathogenic *Legionella* species in a complete water supply system. However, the ecology of *L. pneumophila* and non-*L. pneumophila* species in individual, specific samples were not discussed. The focus of Logan-Jackson et al., 2021 [28] was on understanding the co-occurrence of five of the most-disease relevant *Legionella* species to free-living amoebae, *Naegleria* and *Acanthamoeba*. The improvement of this study to the studies discussed above is investigating only *Legionella* ecology in a complete water supply system (from source to tap) and describing how multiple factors (water age, water quality parameters, etc.) contributes to its occurrence and concentration. 

The goal of this study was to detect and quantify five pathogenic *Legionella* species from a groundwater system including exposure sites such as taps and cooling towers. This study examined the ecology of disease-relevant strains of *Legionella* from the source and treated water system. The following objectives were pursued: (i) quantification of total *Legionella* spp. (23S rRNA), *L. pneumophila*, *L. anisa*, *L. longbeachae*, *L. micdadei*, and *L. bozemanii* in groundwater coming in and out of a reservoir (untreated and treated water storage tanks), the influent pipe at two buildings, the hot and cold-water taps (collected separately) and cooling towers and (ii) exploration of the associations of *Legionella* species with respect to location, temperature, chlorine, conductivity, pH, Heterotrophic Plate Count (HPC), and water age. 

## 2. Materials and Methods

### 2.1. Site Location and Sampling

Forty-two water samples were collected during July, August, and September of 2019 from the reservoir (untreated and treated water storage tanks), two academic/research buildings (Fa, and ERC), and six cooling towers on a research-intensive university campus that runs its own water system. The reservoirs were two different standing storage tanks that contained un-chlorinated water and chlorinated water. Thus, the building water supply secondary disinfectant was free chlorine. The reservoir and cooling towers were sampled six times and the buildings were sampled three times; each sampling replicate were collected on different days. The untreated and treated water storage tanks were referred to as RES_IN and RES_EF, respectively. At the time of sampling, buildings Fa and ERC construction years were 1948 and 1986, respectively. The pipe material for building Fa 75% galvanized and 25% copper. Building ERC pipe material was 50% galvanized and 50% copper. The average monthly water usage (five- year average) and distance from the effluent pipe of the reservoir for building Fa were 172,993 L/month and 4.7 km and for building ERC were 738,533 L/month and 19.4 km. The building size for building Fa was 7118 m^2^ with two floors and for building ERC was 11,896 m^2^ with one floor. The building name description of each building was abbreviated to protect the privacy of the site location. The water samples collected for this study are the same samples from Logan-Jackson et al., 2021 and some of these results in Tables 1 and 4 are available from a previously published paper [28]. 

A ten-liter grab or composite sample was collected from each location to obtain water that was coming directly from untreated ground water, treated (disinfected) water, buildings influent pipe and potable water, and cooling towers. The groundwater source had ~20 production wells, which are completed at a depth of approximately 435 feet. The wells drew water from an aquifer and the un-treated ground water is delivered directly to an untreated storage tank. The influent reservoir pipe (RES_IN) seeds water into another storage tank, where the water is treated before pumped to the buildings. The water treatment was solely chlorine. The effluent reservoir pipe (RES_EF) distributes water into the buildings service lines and cooling towers on a university campus. Water was collected from the source to tap to understand how the water age impacts the microbial occurrence and concentration. The water age in this study is presented as hours and it is defined as follows: the time from the ground water wells to RES_IN (4.5 h), RES_IN to RES_EF (3.4 h), RES_EF to the influent pipe of building Fa and ERC (9.2 h and 20.8 h, respectively), and the cooling towers (23.2 h). 

Ten liters of grab sample were collected from the RES_IN and RES_EF, both building’s influent, as well as from the cooling towers. RES_IN and RES_EF samples were collected directly from the influent and effluent pipes of the reservoir, respectively. Both building’s influent samples were collected directly from the buildings service lines. For the buildings (Fa and ERC) cold- and hot-water potable samples, 10 L composite sampling was based on the number of taps per floor. The building composite samples comprised of the first draw samples from all locations investigated. The reasoning behind this sampling scheme was to determine the percent positive for the entire building and not a single location in the building. The point of use sample locations were sink faucets and showerheads. Building Fa had two floors with two and three sinks on the first and second floor, respectively. ERC had one floor with 11 sinks and two shower sampling locations. The cold and hot-water faucets were composited separately to evaluate the different water systems. For the potable water samples, building Fa had 2–10 L composite samples per floor (4–10 L composite samples total), while ERC only had 2–10 L composite samples total. To make a total of 4–10 L composite samples in building Fa, 5- and 3.3-L composite samples were collected on floors one and two, respectively. The reason buildings Fa and ERC had 4– and 2–10 L composite samples were to determine the building by compositing the tap (cold and hot) per floor, if any. To composite 10 L in building ERC, 0.77 L of water was collected from each cold and hot water point of use, separately. Each carboy contained 10 mL of 10% sodium thiosulfate to neutralize residual chlorine. Per sampling day, building Fa had a total of five samples: one for the influent sampling location and four composite samples (cold- and hot-water). Per sampling day, building ERC had a total of three samples: one for the influent sampling location and two composite samples (cold- and hot-water). There were several cooling towers on the research institution, including on the top of buildings and the power plant, but it was decided to only sample six cooling towers from the power plant. In detail, 12 samples were collected from the reservoir (six, RES_IN and six, RES_EF), 15 from building Fa, nine from building ERC, and six from the cooling towers, all replicates were collected on different days. 

### 2.2. Chemical-Physical and Microbiological Analysis

A 300 mL sample was collected for physiochemical parameters. During sampling, the temperature and chlorine residuals (total and free) were measured using calibrated thermometers and the Test Kit Pocket Colorimeter II (HACH^®^, Loveland, CO, USA) according to the manufacturer’s instructions. After sampling, conductivity, pH, and turbidity were measured at the laboratory according to the manufacturers’ instructions using a Russell RL060C Portable Conductivity Meter (Thermo Scientific, Waltham, MA, USA), UltraBasic pH meter (Denver Instrument, Bohemia, NY, USA), and a Turbidity Meter code 1970-EPA (LaMottee Company, Chestertown, MD, USA). Conductivity is an important measurement to determine the impurity level of dissolved substances, chemicals and minerals, and pH is an important indicator to determine how the water is changing chemically. Turbidity is a critical measurement that determines the clarity of a water sample. After collecting the water samples, all samples were placed on ice and transported to the laboratory and immediately processed for ultrafiltration (described below) and HPCs. HPC is a procedure for measuring live bacteria in water samples; it helped to measure the changes in culturable bacteria from source to tap. All samples were tested for HPC analyses using membrane filters (47 mm diameter, 0.45 μm pore size; PALL Corporation, Port Washington, NY, USA) on m-HPC agar (Becton, Dickinson and Company, Difco, Detroit, MI, USA). The plates were incubated for 48 ± 2 h at 37 °C, then enumerated for colony-forming units. In addition to HPCs, all samples were also tested for total and fecal coliforms using Colilert (IDEXX Laboratories, Westbrook, ME, USA). 

### 2.3. Water Sample Processing

All 42–10 L samples were processed using a single Asahi REXEED-25S dialysis filter (Dial Medical Supply, Chester Springs, PA, USA). Each filter was pretreated with 0.01% of sodium hexametaphosphate—used to trap microbial material onto the filters. Each filter was used in a dead-end ultrafiltration approach. A high-pressure single-use elution fluid canister (INNOVAPREP LLC, Drexel, MO, USA) was used to concentrate the 10 L to ~50 mL, and each ultrafiltration concentrate was split into several 10 mL subsamples for preservation—to reduce the need for several freeze/thaw cycles. There were 5–10 mL subsamples. 

### 2.4. DNA Extraction and Quantitative Detection of Legionella Species Using Droplet Digital PCR

Each 10 mL subsample (one/sample) was filtered on to a polycarbonate filter (47 mm diameter, 0.45 μm pore size; Whatman, Kent, UK) inside of a sterilized 47 mm diameter magnetic filter funnel (PALL Corporation, Port Washington, NY, USA). The filter containing the environmental contents was then transferred to a 2.0-mL polypropylene screw cap tube (VWR, Radnor, PA, USA) containing 0.3 g of 212–300 μm acid-washed glass beads (Sigma, St. Louis, MO, USA) for DNA extraction. DNA was extracted by adding 590 μL of AE buffer (Qiagen, Hilden, Europe) to each screw-cap tube. The filters with the glass beads were then bead milling using a FastPrep-24™ 5G Instrument MP Biomedicals (VWR). Each sample was milled at 6000 rpm for one minute, followed by centrifugation at 12,000× *g* for one minute. The supernatant (~400 μL) was then transferred to a new sterilized microcentrifuge tube and centrifuged at 12,000× *g* for three minutes to pellet any remaining debris. Extracted DNA was eluted (~350 μL) into a final sterilized microcentrifuge tube. The eluted DNA was aliquoted into five subsamples which contained ~60 μL of the eluted nucleic acid and stored at −80 °C. Aliquoting the eluted DNA help to reduce the need for several freeze/thaw cycles in the event the sample needed to be re-ran. One aliquot per water sample was later used for PCR analysis (ddPCR analysis occurred within 30 days of extraction). Ten milliliters of phosphate-buffer water were filtered and functioned as a filtration blank. 

Droplet digital PCR technology was performed according to the manufacturer’s instructions to analyze each sample for *Legionella.* All positive controls were obtained from American Type Culture Collection (ATCC, Manassas, VA, USA). Each sample was analyzed for *Legionella* spp. (23S rRNA), and five pathogenic species (*L. pneumophila* [ATCC No. 33152], *L. anisa* [ATCC No. 35292], *L. micdadei* [ATCC No. 33218], *L. bozemanii* [ATCC No. 33217], and *L. longbeachae* [ATCC No. 33462]). All primers and probes were ordered from Eurofins Genomics co. (Louisville, KY, USA). The primers and probes used in this study are listed in Supplementary Materials Table S1. Per assay, there was one ddPCR run with triplicate biological (sample collection) and technical replicates (same extraction). Three duplex reactions were performed for this study: the first assay comprised of *Legionella* spp. (23S rRNA) and *L. pneumophila*; the second assay consisted of *L. micdadei* and *L. anisa*; the third assay contained *L. bozemanii* and *L. longbeachae*. For each assay: sterilized molecular grade-water (without template) served as a no-template control to detect environmental contamination and phosphate-buffer water (PBW) functioned as an experimental control, this helps to determine if the PBW introduced contamination. There were five microbial DNA positive controls: *L. pneumophila*, *L. micdadei*, *L. anisa*, *L. bozemanii*, and *L. longbeachae* used to verify the efficiency of the assay. The negative and positive controls listed above served as experimental controls. Sample results were only considered for analysis when the reader accepted 10,000 or more droplets as part of the quality control, and unknown samples with three or more positive droplets per well were considered a true positive. The limit of detection for the assays were 1.3 log per 100 mL. *Legionella* species were detected and quantified when the value was at or above 1.3 log per 100 mL. Specific details of ddPCR and the definition of the limit of detection and quantification is presented in the Supplementary Information.

Each amplification ddPCR reaction mixture consisted of 2X supermix (no dUTP) (Bio-Rad Laboratories, Hercules, CA, USA), mixed with a final concentration of 900 nM forward and reverse primers and 250 nM probes and up to 330 ng of DNA template in a final volume of 20 μL. Droplets were generated according to the manufacturer’s instructions using a QX200 Droplet Generator. Endpoint PCR was performed in a T100 Thermal Cycler (Bio-Rad Laboratories, Hercules, CA, USA) and the cycling protocol was as follows: 95 °C for 10 min, followed by 40 cycles of 94 °C for 30 s and 57 °C for 1 min with a final 10 min cycle at 98 °C for 10 min. The plate was then cooled for ~30 min, and droplets were then read using a QX200 droplet reader (Bio-Rad QX200^TM^ Droplet Digital PCR System, Hercules, CA, USA). 

### 2.5. Statistical Analysis

Descriptive statistics were conducted in GraphPad Prism 8 software (GraphPad Software, San Diego, CA, USA). Statistical analysis, including One-way ANOVA, Pearson Correlation, and simple linear regression, were used to determine the significance of the findings. The biological data were expressed as the geometric mean with geometric deviation, and chemical data were shown as arithmetic means with standard deviation. A geometric mean for each sample was calculated using all values from technical and biological replicates. Sample concentrations were transformed from gene copies (GC)/100 mL into Log_10_ GC/100 mL for statistical analysis [28]. Statistical results were interpreted at the level of significance *p* < 0.05. 

## 3. Results

### 3.1. Characterization and Concentrations of Legionella 23S rRNA and Five Pathogenic Legionella Species 

*Legionella* spp. (23S rRNA) was found in 100% (42/42) of water samples at concentrations ranging from 2.2 to 4.5 Log_10_ GC/100 mL. *Legionella pneumophila* was found in 57% (24/42) of the water samples, followed by *L. bozemanii* 52% (22/42), *L. longbeachae* 36% (15/42), *L. micdadei* 23% (10/42), *L. anisa* 21% (9/42) at geometric mean concentrations of 1.7, 1.7, 1.4, 1.6 and 1.7 Log_10_ GC/100 mL, respectively (Table 1). Some of the results in Table 1 are also available in a previously published paper [28]. Table 1 is adapted with permission from ref. [28]. (2021, Alshae, R. Logan-Jackson).

Out of 42 samples, 16% (7/42) of them only contained *L. pneumophila* and another 14% (6/42) of the samples contained only one of the five *Legionella* species (Supplementary Information Table S2). The co-occurrence of *L. pneumophila* with other *Legionella* species is considered in greater detail in the Supplemental Information Table S2. Table 2 presents the percentages of co-contamination with multiple *Legionella* species. 

### 3.2. Detection of 23S rRNA and Five Legionella Species from Groundwater Source to the Taps in the Buildings, to the Cooling Towers

**Figure 1:**
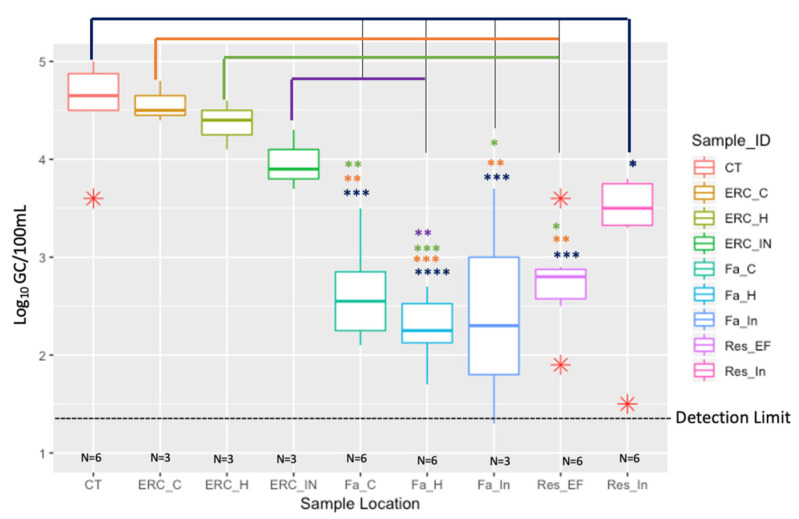
Comparison of *Legionella* spp. (23S rRNA) in the reservoir (RES_IN and RES_EF), buildings: Fa and ERC, and the Cooling Towers (CT). The water age (h) in Res_In (4.5 h), Res_EF (3.4 h), Fa (9.2 h), ERC (20.8 h), and CT (23.2 h). Description names are as follows: Res_In: Reservoir Influent; Res_Ef: Reservior Effluent; Fa_In: Fa Influent; Fa_H: Fa Hot-water tap; Fa_C: Fa Cold-water tap; ERC_In: ERC Influent; ERC_H: ERC Hot-water tap; ERC_C: ERC Cold-water tap; CT: Cooling Towers. The median of each measure is indicated by the thick colorful bar in each respective box; the first and third quartiles are represented by the bottom and top of the box, respectively; the red asterisks (*) show depicts the outliers within three sample types. The different asterisk (*, **, ***, ****) colors represent the significant difference between sampling location and is as follows: blue: CT; orange: ERC_C; green: ERC_H; purple: ERC_IN. Detection limit was 1.3 Log_10_ GC/100 mL.

The concentration of total *Legionella* spp. (23S rRNA) in the influent pipe of the reservoir (RES_IN) was 3.1 Log_10_ GC/100 mL and in the effluent pipe of the reservoir (RES_EF) the concentration decreased to 2.7 Log_10_ GC/100 mL. The Log_10_ GC/100 mL of total *Legionella* spp. (23S rRNA) of building Fa [in the influent water pipe, the cold- and hot-water taps] was about the same as the reservoir effluent, all four sampling locations ranged from 2.2 to 2.7 Log_10_ GC/100 mL (Figure 1). It is interesting to note that the similar concentrations of total *Legionella* spp. between building Fa and the reservoir may be due to the fact this building is closest to the reservoir (water age is 9.2 h) relative to the building ERC. Building ERC had a water age of 20.8 h, and the concentration of total *Legionella* spp. significantly increased (Figure 1). Total *Legionella* spp. (23S rRNA) concentrations in building ERC detected in the influent water pipe, hot- and cold-water taps were 4.0, 4.3 and 4.5 Log_10_ GC/100 mL, respectively (Figure 1). The cooling towers (4.5 Log_10_ GC/100 mL) had similar concentrations of total *Legionella* spp. (23S rRNA) compared to building ERC, once again significantly (*p* = 0.0003) higher than the reservoir effluent (2.7 Log_10_ GC/100 mL) (Figure 1). These data suggests that both the water age and more importantly building water management contributes to the growth of *Legionella* species.

Overall, the geometric mean concentrations of total *Legionella* spp. (23S rRNA) in the cooling towers and in the ERC building (both influent, hot- and cold-water taps) were statistically higher than what was found in the influent and effluent of the reservoir, influent, cold- and hot-water taps of building Fa (Figure 1). The *p* values showing the most significance were the cooling towers compare to the reservoir effluent and building Fa. This significant pattern was also true for the ERC building compared to building Fa and the reservoir (Figure 1). The detailed description of the ANOVA results are as follows: CT vs. Res_In, (0.0156); CT vs. Res_EF (0.0003); CT vs. Fa_In (0.0006); CT Vs Fa_H (<0.0001); CT vs. Fa_C (0.0001); ERC_C vs. Res_EF (0.0043); ERC_C vs. Fa_In (0.0036); ERC_C vs. Fa_H (0.0002); ERC_C vs. Fa_C (0.0020); ERC_H vs. Res_EF (0.0152); ERC_H vs. Fa_In (0.0107); ERC_H vs. Fa_H (0.0007); ERC_H vs. Fa_C (0.0074); ERC_In vs. Fa_H (0.0091) Table 3. The five *Legionella* species concentration data did not show a clear trend relative to water age; thus, it was decided to present the data in a table format (Table 1). 

Specific pathogenic *Legionella* species were detected in the reservoir (influent and effluent), the buildings, and the cooling towers. The geometric means with non-detects used at the detection limit are presented in the paragraphs below. *Legionella bozemanii*, *L. micdadei*, and *L. pneumophila* were detected in the influent of the reservoir (Res_IN) at geometric mean concentrations of 1.5, 1.5, 1.6 Log_10_ GC/100 mL, respectively. *Legionella pneumophila* and *L. bozemanii* were detected in the effluent of the reservoir at geometric mean concentrations of 1.8 and 1.7 Log_10_ GC/100 mL. *Legionella longbeachae*, *L. pneumophila*, and *L. micdadei* were detected in the influent water pipes of buildings Fa at geometric mean concentration of 1.5, 1.8, and 1.6 Log_10_ GC/100 mL, respectively. All three species concentrations decreased at the taps in building Fa (Table 1). *Legionella micdadei*, *L. bozemanii*, *L. pneumophila*, and *L. longbeachae* were detected in the influent water pipes of the ERC building at geometric mean concentrations of 1.6, 1.6, 1.4, and 1.4 Log_10_ GC/100 mL, respectively (Table 1). All four species concentrations either stabilized (same concentration) or decreased at the taps in building ERC (Table 1).

Most of the pathogenic *Legionella* species were much more prevalent and found in cooling towers at much higher concentrations. In the cooling towers, *L. bozemanii* had the highest concentration at 3.0 Log_10_ GC/100 mL followed by *L. pneumophila* (2.8), *L. micdadei* (2.4), *L. anisa* (2.1), and *L. longbeachae* at 1.5 Log_10_ GC/100 mL (Table 1 and Table 3).

### 3.3. Water Quality Parameters

The water quality characteristics of the reservoir, the buildings, and the cooling towers are presented in Table 4. 

Water temperature in the reservoir (influent and effluent) ranged from 11.6 to 12.3 °C; the free chlorine residual in the reservoir influent was 0 mg/L, and in the reservoir effluent, the average value was 0.64 mg/L (water quality averages shown in Table 4). The conductivity ranged from 620 to 1032 μS/cm and the turbidity ranged from 1.08 to 9.55 NTU; the pH ranged from 7.1 to 7.4. The HPCs in the reservoir influent ranged from 1.50 × 10^1^ to 7.80 × 10^1^ CFU/100 mL, and decreased in the reservoir effluent, and ranged from 1.0 to 6.0 CFU/100 mL. The water quality parameters between the building influents of Fa and ERC were statistically different from each other. The water temperatures, turbidity, pH, and HPC (cold and hot separate samples) on both floors in building Fa were statistically different from building ERC (Table 4). Overall, the water temperature at the effluent of the reservoir was 11.9 °C but increased at both Fa (26.8 °C) and ERC (31.5 °C) building influent pipe—suggesting that water age is a factor in the water quality parameters as it relates to the pipe distance within each building incoming water and not at the cold- and hot-water taps. Some of the results in Table 4 are also available in a previously published paper [28]. Table 4 is adapted with permission from ref. [28]. (2021, Alshae, R. Logan-Jackson).

A higher variance of all the water quality parameters was noted in the cooling towers (Table 4). Interestingly, total coliforms and *E. coli* were also seen in the cooling towers at 17.3 and 666.6 MPN/100 mL, respectively (Table 4). The cooling towers receive water from the effluent of the reservoir; thus, the contamination of *E. coli* may be due to birds—this hypothesize was thought of because the cooling water is directly exposed to the atmosphere. Biocides used in the cooling tower are in rotation with different chemicals: glutaraldehyde, a mixture of polyethylene glycol and 2,2-Dibromo-3-nitrilopropionamide, as well as sodium hypochlorite (chlorine was used separately from the organic biocides). However, these different types of substances/mixtures did not always control fecal indicator and HPC bacteria but may be decreasing cultivatable *Legionella* (as the utility responsible for the cooling towers does monitor *L. pneumophila* with culture techniques by a state laboratory).

## 4. Discussion

While there have been several studies that revealed the differences of total *Legionella* spp. comparing various exposure sites [20,29,30,31,32,33], only a couple of studies have examined how the hydraulic retention time (water age) influenced *Legionella* spp. in building water systems [34,35]. Nguyen et al., 2012 [34] compared four tap water sites in an undisclosed location where one site lacked a toilet (thus water use was decreased) and found no difference in *Legionella* species detected. Rhoads et al., 2016 [35] surveyed four buildings, three buildings were green compared to a conventional house. The hydraulic retention time for each house/ building was ~1, 2.7, 8 and 30–180 days; quantitative PCR (qPCR) data showed that *Legionella* spp. (23S rRNA) were mostly detected in the green buildings (water ages were 2.7 and 30–180 days) relative to the conventional buildings (water age of 1 and day/s) [35]. 

Low water usage can increase water age as defined by the length of time the water sits in the premise plumbing; this can be influenced by low number of occupants in a portion of the building, water conservation features, or even the low flow features. However, day-to-day water stagnation in complex water systems is a challenging parameter to measure. Nguyen et al. (2012) [34] and Rhoads et al. (2016) [35] determined water age by water usage patterns within premise plumbing systems. The study described herein evaluated the water age as it related to pipe mileage from the reservoir to the premise for buildings Fa and ERC, which are closest and furthest from the reservoir, respectively. By collecting 10 L samples the goal was to understand how water age contributes to the growth of *Legionella* throughout a building as opposed to an individual tap/s. Building design and operation contributes to water age at various taps throughout an entire building; thus, the authors believed that collecting 10 L composite samples in a building would help to compare the water ages of buildings that are structurally different to the water source and cooling towers. Building ERC had high levels of *Legionella* 23S rRNA despite high water use (five-year average: 738,533 L/month compared to FA five-year average of 173,993 L/month) due to use of a cooling tower, which was located on top of the premise—the higher water usage patterns did not impact the *Legionella* concentrations. Building Fa had a lower water usage pattern, and lacked a cooling tower, but had less hydraulic retention time in the distribution system (closest to the reservoir); thus, less time in the distribution system contributed to lower levels of *Legionella* spp. (23S rRNA) at every sampling location inside the building. These data suggests that the length of pipe and the distance from the water source influenced the premise water age and not the water usage patterns (which is influenced by occupants). The other water quality parameters including pH, HPC, turbidity, and temperature were also different between the two buildings, however these characteristics were not predictive in terms of the increase of *Legionella* observed at the taps. The regression analysis data suggested that the water age is driving the relationship between water quality parameters (HPCs, pH, temperature, and turbidity) and *Legionella* spp. (23S rRNA) and not the individual indicators (data not shown). However, this observation from the regression analysis will need to be further elucidated with a more extensive data set: such as increasing the total number of samples, sampling and analyzing the biofilm, and verifying the ddPCR results with DNA sequencing. Overcoming such limitations would increase this study significance. Nonetheless, the regression analysis further emphasizes that it is a building water issue—the building management and the distance from the water source influenced the premise water age which impacted the water quality in buildings Fa and ERC, causing these two buildings to be distinctly different

*Legionella* species that are mostly associated with human disease are *L. pneumophila*, *L. micdadei*, *L. bozemanii*, *L. longbeachae*, and *L. anisa* [6]. In the U.S., there were approximately 7500 and 10,000 Legionnaires’ Disease cases reported in 2017 and 2018, respectively [36]. The increasing trend of Legionnaires’ Disease cases are primarily attributed to *L. pneumophila* [37] which is thought to account for 95% of the cases. However, *L. anisa*, *L. micdadei*, *L. bozemanii*, and *L. longbeachae* may pose as great a risk as *L. pneumophila*—or more than previously recognized [6]. In several studies *L. anisa* species appeared to be more prevalent in environmental water samples [38,39,40] at levels as high as 3.0 X 10^4^ GC/100 mL [41]. Health Departments should consider Legionnaires’ Disease from other *Legionella* species as these species are also found in building water systems and cooling towers [25,28,42,43,44,45]. As shown here *Legionella* pathogenic species are contributing to approximately 0.1 to 10% of the *Legionella* present at the taps and cooling towers and in some cases the concentrations of the specific *Legionella* species were high enough to be of some concern (*L. bozemanii*—3.0 Log_10_ GC/100 mL). In large community-wide outbreaks of Legionnaires’ Disease such as in Flint, Michigan [46,47] where multiples species could possibly be causing disease, only *L.* *pneumophila* is looked for and thus disease caused by other species would go underestimated. 

In this study, the concentrations of the specific *Legionella* pathogenic species ranged from 1.1 to 3.0 Log_10_ GC/100 mL (geomean: 1.6 Log_10_ GC/100 mL) at the exposure sites. Previous risk assessments suggest that the level which equates to approximately 10^−4^ annual goal for drinking water safety is around 5.0 Log_10_ CFU/L (4.0 Log_10_ CFU/100 mL) for faucets and 3.0 Log_10_ CFU/L (2.0 Log_10_ CFU/100 mL) for showers [48]. Thus, these concentrations at the exposure sites are near the level of the acceptable annual goal— assuming these other pathogenic species are 100% cultivable and have similar dose–response characteristics (for example, the number and expression of virulence genes) as *L. pneumophila.* This assumption is also based on the fact the concentrations presented herein would be similar throughout the year. 

There are guidelines and regulations for the control of *Legionella* in cooling tower systems. For example, in the U.S., guidance is provided by ASHRAE 188 standard and American Industrial Hygiene Association [49,50], while some states (e.g., New York) have a mandatory monitoring scheme which is based on culturing *Legionella* that grows on a selective media [51]. The concentration of concern is equal to or greater than 10^3^ CFU/mL (10^5^ CFU/100 mL) and this would require corrective action [50]. *Legionella* spp. (23S rRNA), and pathogenic species concentrations (listed above) were near the safety levels established in the AIHA guidelines [50]. While it understood that concentrations of *Legionella* by the culture method (CFU) cannot be directly compared to molecular methods (PCR), it is worth noting that *Legionella* spp. (23S rRNA), and pathogenic species concentrations were 4.5 and 3.0 Log_10_ GC/100 mL, respectively by ddPCR. Although, ddPCR could be evaluating non-infectious bacteria, it could also be enumerating bacteria inside its host cell [52]. 

*Legionella* species occurrence and concentration may be underestimated by utilizing a small grab sample (0.25 L or 1 L) and quantifying using culture techniques. It will be critical to understand whether these other *Legionella* disease relevant species at such concentrations (i.e., *L. bozemanii* 3.0 Log_10_ GC/100 mL) are also associated with risk— as the cooling towers are in the ideal temperature range (25 °C–45 °C, [53]) for these species to proliferate. Thus, a rapid PCR monitoring scheme —using a 10 L composite sample—could result in improved strategies to control the amplification of disease relevant *Legionella* species (*L. pneumophila*, *L. anisa*, *L. micdadei*, *L. bozemanii*, and *L. longbeachae*) in taps and cooling towers to holistically understand the building water quality rather than individual taps. While this study revealed information about how the water age and building water quality impacted the occurrence and concentration of *Legionella* species, this study would benefit from a more extensive, year-long data set to achieve a higher significance. It is also important to note that the temperature data were not taken from the water heater set point or the recirculation loops in the two buildings; thus, this study could also benefit from a systematic understanding on how different the water quality is as it relates to the cold- and hot-water systems. 

## 5. Conclusions

Overall, water age, as defined in this study, is only one of multiple factors that plays a role in the *Legionella* spp. colonization of buildings and cooling towers, as seen in the ERC building. Water age is an important factor that contribute to the occurrence and the abundance of *Legionella* species but is not the most important factor. For example, water management in the building is the most critical factor that contributes to the growth of *Legionella* species. Critical building factors such as infrequent water usage, water quality parameters, or the plumbing design of a building may create growth and niches for *Legionella.* Aside from building management, water quality in buildings, especially the buildings that’ are furthest away from water treatment may be challenged, thus, further information by water utilities on hydraulic retention times and disinfection boosters may be needed. Concentrations increasing above 100 GC/L could be targeted for remediation. Distribution and premise plumbing systems remain a source of risk from several pathogenic *Legionella* species. It is likely that disease associated with *L. micdadei*, *L. bozemanii*, *L. longbeachae*, and perhaps *L. anisa* are underestimated and more information on culturability and viability are warranted. 

Since 2009, *L. pneumophila* has been on the United States Environmental Protection Agency (USEPA) Candidate Contaminant List (CCL); thus, there are reasons to believe that there should be federal regulations for monitoring and controlling this primary water-related bacterium. Ultimately, a routine monitoring scheme would help to target better management approaches to decrease the morbidity and mortality rate of Legionnaires’ Disease—caused by *L. pneumophila*, and other species from common exposure sites (hot-water taps and especially cooling towers) where conditions are favorable for their proliferation, and where *Legionella*-containing aerosols are generated. 

## Figures and Tables

**Table 1:** Geometric mean concentrations of general *Legionella* (23S rRNA) and five specific *Legionella* species collected from the drinking water system (RES_IN, RES_EF, Fa_IN, Fa_Taps, ERC_IN, ERC_Taps) and the cooling towers (CT).

*Legionella* Species ^b^	CT (*n* = 6)	ERC Taps (Cold *n* = 3) [Hot *n* = 3]	ERC_IN (*n* = 3)	Fa_Taps (Cold *n* = 6) [Hot *n* = 6]	Fa_IN (*n* = 3)	Res_EF (*n* = 6)	Res_In (*n* = 6)
*Legionella* spp. (23S rRNA) (%+) [%+]	100% (6/6)	(100%: 3/3) [100%: 3/3]	100% (3/3)	(100%: 6/6) [10%: 6/6]	100% (3/3)	100% (6/6)	100% (6/6)
*Legionella* spp. (23S rRNA) Geomean (Log _10_GC/100 mL)	4.5	(4.5) [4.4]	4.0	(2.6) [2.2]	2.2	2.7	3.1
*L. pneumophila* (%+) [%+]	83% (5/6)	(100%: 3/3) [0%: 0/3]	33% (1/3)	(33%: 2/6) [33%: 2/6]	67% (2/3)	83% (5/6)	83% (5/6)
*L. pneumophila* Geomean (Log _10_GC/100 mL)	2.8	(1.4) [ND]	1.4	(1.4) [1.6]	1.8	1.8	1.6
*L. micdadei* (%+) [%+]	33% (2/6)	(0%: 0/3) [66%: 2/3]	67% (2/3)	(16%: 1/6) [0%: 0/6]	67% (2/3)	0% (0/6)	17% (1/6)
*L. micdadei* Geomean (Log _10_GC/100 mL)	2.4	(ND ^a^) [2.5]	1.6	(1.1) [ND ^a^]	1.6	ND ^a^	1.5
*L. bozemanii* (%+) [%+]	100% (6/6)	(0%: 0/3) [33%: 1/3]	100% (3/3)	(16%: 1/6) [16%: 1/6]	0% (0/3)	100% (6/6)	67% (4/6)
*L. bozemanii* Geomean (Log _10_GC/100 mL)	2.9	(ND ^a^) [1.8]	1.6	(1.4) [1.8]	ND ^a^	1.7	1.5
*L. longbeachae* (%+) [%+]	50% (3/6)	(0%: 0/3) [33%: 1/3]	100% (3/3)	(66%: 4/6) [50%: 3/6]	33% (1/3)	0% (0/6)	0% (0/6)
*L. longbeachae* Geomean (Log _10_GC/100 mL)	1.5	(ND ^a^) [1.7]	1.4	(1.2) [1.6]	1.5	ND ^a^	ND ^a^
*L. anisa* (%+) [%+]	67% (4/6)	(0%: 0/3) [0%: 0/3]	0% (0/3)	(16%: 1/6) [66%: 4/6]	0% (0/3)	0% (0/6)	0% (0/6)
*L. anisa* Geomean (Log _10_GC/100 mL)	2.1	(ND ^a^) [ND ^a^]	ND ^a^	(1.1) [1.6]	ND ^a^	ND ^a^	ND ^a^

^a^ ND: No Detect; Detection limit is 1.3 Log_10_ GC/100 mL. ^b^ The building samples were composites; thus, the *Legionella* concentrations represent composite concentration and not individual tap concentration.

**Table 2:** Number of samples with no-detects, single and multiple *Legionella* species.

Number of Different *Legionella* spp.	Percent Positive %+ (Sample Positive/Total Number of Samples)
0	7 (3/42)
1	30 (13/42)
2	30 (13/42)
3	23 (10/42)
4	4 (2/42)
5	2 (1/42)

**Table 3:** Detailed description of the ANOVA results for the significant pairs shown in Figure 1.

No. of Pairs	Pairs	*p*-Value	Remarks
1	CT vs. RES_IN	0.0156	Significant
2	CT vs. RES_EF	0.0003	Highly significant
3	CT vs. Fa_IN	0.0006	Highly significant
4	CT vs. Fa_H	<0.0001	Highly significant
5	CT vs. Fa_C	0.0001	Highly significant
6	ERC_C vs. RES_EF	0.0043	Very significant
7	ERC_ vs. Fa_IN	0.0036	Very significant
8	ERC vs_Fa_H	0.0002	Highly significant
9	ERC_C vs Fa_C	0.0020	Very significant
10	ERC_H vs. RES_EF	0.0152	Significant
11	ERC_H vs. Fa_IN	0.0107	Significant
12	ERC_H vs. Fa_H	0.0007	Highly Significant
13	ERC_H vs. Fa_C	0.0074	Significant
14	ERC_IN vs. Fa_H	0.0091	Significant

**Table 4:** Water quality parameters of the reservoir (influent and effluent), the influent, hot- and cold-water (all three sample types were collected on the same day, but replicates were collected on different days) in buildings (Fa and ERC), and the CT. Sample collection dates are as follows: reservoir (influent and effluent), 15, 23 and 29 July and 6, 13 and 20 August; influent, hot-, and cold-water in building Fa, 12 August and 3, 16 September; influent, hot-, and cold-water in building ERC, 19 August, and 9, 23 September; CT, 25, 31 July and 7, 14 and 21 August; two cooling tower water samples were collected on the 21st.

Temperature (°C)	Total Chlorine (mg/L)	Free Chlorine (mg/L)	Turbidity (NTU)	pH	Conductivity (mS)	HPC (CFU/100 mL)	Total Coliforms (MPN/100 mL)	*E. coli* (MPN/100 mL)
**Reservoir Influent (*n* = 6)**								
12.1	0	0	4.1	7.2	851	3.52 × 10^1^	<1	<1
**Reservoir Effluent (*n* = 6)**								
11.9	0.64	0.33	3.85	7.2	855	2.10 × 10^0^	<1	<1
**Building Fa Influent (*n* = 3)**								
26.8	0.41	0.35	8.4	7.3	897	8.57 × 10^4^	<1	<1
**Building Fa 1 st Floor Cold; *n* = 3** **(Hot Taps; *n* = 3)**								
26.7 (28.6)	0.16 (0.04)	0.14 (0.02)	3.06 (0.53)	7.2 (7.1)	867 (815)	1.02 × 10^4^ (7.3 × 10^3^)	<1 (<1)	<1 (<1)
**Building Fa 2nd Floor Cold; *n* = 3** **(Hot Taps; *n* = 3)**								
26.8 (28.8)	0.05 (0.02)	0.03 (0)	3.37 (0.67)	7.0 (6.9)	856 (822)	2.00 × 10^4^ (3.15 × 10^3^)	<1 (<1)	<1 (<1)
**Building ERC Influent (*n* = 3)**								
31.5	0.31	0.20	12.5	7.4	883	4.32 × 10^5^	<1	<1
**Building ERC 1 st Floor Cold (*n* = 3)** **(Hot Taps; *n* = 3)**								
23.5 (24.5)	0.09 (0.04)	0.03 (0)	5.97 (6.27)	7.6 (7.5)	866 (847)	4.38 × 10^5^ (6.80 × 10^5^)	<1 (<1)	<1 (<1)
**CT (*n* = 6)**								
25.3	0.49	0.08	1.94	8.2	2564	2.35 × 10^7^	666.6	17.3

## Supplementary Materials

The following are available online at https://www.mdpi.com/article/10.3390/microorganisms10010081/s1, Table S1. General and specific *Legionella* primers and probes. 23S pan-*Legionella* is conserved by all species of *Legionella;* but the probes were uniquely designed to specifically identify each species: *L. micdadei*, *L. anisa*, *L.bozemanii*, and *L. longbeachae*. ddPCR Information. Table S2. Raw data of Five Disease Relevant *Legionella* species. References [54,55] are cited in the Supplementary Materials.

## References

[1] Fraser D.W., Tsai T.R., Orenstein W., Parkin W.E., Beecham H.J., Sharrar R.G., Harris J., Mallison G.F., Martin S.M., McDade J.E. (1977). Legionnaires’ disease: Description of an epidemic of pneumonia. N. Engl. J. Med..

[2] Brenner D.J., Steigerwalt A.G., McDade J.E. (1979). Classification of the Legionnaires’ disease bacterium: *Legionella pneumophila*, genus novum, species nova, of the family Legionellaceae, familia nova. Ann. Intern. Med..

[3] Zeng L.Z., Liao H.Y., Luo L.Z., He S.S., Qin T., Zhou H.J., Li H.X., Chen D.L., Chen J.P. (2019). An Investigation on the Molecular Characteristics and Intracellular Growth Ability among Environmental and Clinical Isolates of *Legionella pneumophila* in Sichuan Province, China. Biomed. Environ. Sci. BES.

[4] Brady M.F., Sundareshan V. (2019). Legionnaires’ Disease (*Legionella* Infection). StatPearls.

[5] Waldron P.R., Martin B.A., Ho D.Y. (2015). Mistaken identity: *Legionella micdadei* appearing as acid fast bacilli on lung biopsy of a hematopoietic stem cell transplant patient. Transpl. Infect. Dis..

[6] Muder R.R., Victor L.Y. (2002). Infection Due to *Legionella* Species Other Than *L**. pneumophila*. Clin. Infect. Dis..

[7] Sanchez M.C., Sebti R., Hassoun P., Mannion C., Goy A.H., Feldman T., Mato A., Hong T. (2013). Osteomyelitis of the patella caused by *Legionella anisa*. J. Clin. Microbiol..

[8] Lachant D., Prasad P. (2015). *Legionella micdadei*: A Forgotten Etiology of Growing Cavitary Nodules: A Case Report and Literature Review. Case Rep. Pulmonol..

[9] Miller M.L., Hayden R., Gaur A. (2007). *Legionella Bozemanii* Pulmonary Abscess in a Pediatric Allogeneic Stem Cell Transplant Recipient. Pediatric Infect. Dis. J..

[10] Centers for Disease Control and Prevention *Legionella* (Legionnaires’ Disease and Pontiac Fever). Surveillance Report 2016–2017. https://www.cdc.gov/legionella/health-depts/surv-reporting/2016-17-report-tables/index.html#figure1.

[11] Brunkard J.M., Ailes E., Roberts V.A., Hill V., Hilborn E.D., Craun G.F., Rajasingham A., Kahler A., Garrison L., Hicks L. (2011). Surveillance for waterborne disease outbreaks associated with drinking water—United States, 2007–2008. Morb. Mortal. Wkly. Rep. Surveill. Summ..

[12] United States Geological Survey Summary of Estimated Water Use in the United States in 2015. https://pubs.usgs.gov/fs/2018/3035/fs20183035.pdf.

[13] Department of Environmental Quality Fact Sheet. Groundwater Statistics. https://www.michigan.gov/documents/deq/deq-wd-gws-wcu-groundwaterstatistics_270606_7.pdf.

[14] Brooks T., Osicki R., Springthorpe V., Sattar S., Filion L., Abrial D., Riffard S. (2004). Detection and identification of *Legionella* species from groundwaters. J. Toxicol. Environ. Health Part A.

[15] De Giglio O., Napoli C., Apollonio F., Brigida S., Marzella A., Diella G., Calia C., Scrascia M., Pacifico C., Pazzani C. (2019). Occurrence of *Legionella* in groundwater used for sprinkler irrigation in Southern Italy. Environ. Res..

[16] Valciņa O., Pūle D., Mališevs A., Trofimova J., Makarova S., Konvisers G., Bērziņš A., Krūmiņa A. (2019). Co-Occurrence of Free-Living Amoeba and *Legionella* in Drinking Water Supply Systems. Medicina.

[17] Mapili K., Pieper K.J., Dai D., Pruden A., Edwards M.A., Tang M., Rhoads W.J. (2020). *Legionella pneumophila* occurrence in drinking water supplied by private wells. Lett. Appl. Microbiol..

[18] Johnson C.D., Nandi A., Joyner T.A., Luffman I. (2018). Iron and Manganese in Groundwater: Using Kriging and GIS to Locate High Concentrations in Buncombe County, North Carolina. Ground Water.

[19] Cianciotto N.P. (2015). An update on iron acquisition by *Legionella pneumophila*: New pathways for siderophore uptake and ferric iron reduction. Future Microbiol..

[20] Donohue M.J., O’Connell K., Vesper S.J., Mistry J.H., King D., Kostich M., Pfaller S. (2014). Widespread Molecular Detection of *Legionella pneumophila* Serogroup 1 in Cold Water Taps across the United States. Environ. Sci. Technol..

[21] Llewellyn A.C., Lucas C.E., Roberts S.E., Brown E.W., Nayak B.S., Raphael B.H., Winchell J.M. (2017). Distribution of *Legionella* and bacterial community composition among regionally diverse US cooling towers. PLoS ONE.

[22] Prussin A.J., Schwake D.O., Marr L.C. (2017). Ten Questions Concerning the Aerosolization and Transmission of *Legionella* in the Built Environment. Build. Environ..

[23] Dilger T., Melzl H., Gessner A. (2017). *Legionella* contamination in warm water systems: A species-level survey. Int. J. Hyg. Environ. Health.

[24] Richards C.L., Broadaway S.C., Eggers M.J., Doyle J., Pyle B.H., Camper A.K., Ford T.E. (2018). Detection of Pathogenic and Non-pathogenic Bacteria in Drinking Water and Associated Biofilms on the Crow Reservation, Montana, USA. Microb. Ecol..

[25] Lesnik R., Brettar I., Höfle M.G. (2016). *Legionella* species diversity and dynamics from surface reservoir to tap water: From cold adaptation to thermophily. ISME J..

[26] Tsao H.-F., Scheikl U., Herbold C., Indra A., Walochnik J., Horn M. (2019). The cooling tower water microbiota: Seasonal dynamics and co-occurrence of bacterial and protist phylotypes. Water Res..

[27] Pereira R.P.A., Peplies J., Brettar I., Höfle M.G. (2017). Development of a genus-specific next generation sequencing approach for sensitive and quantitative determination of the *Legionella* microbiome in freshwater systems. BMC Microbiol..

[28] Logan-Jackson A., Rose J.B. (2021). Cooccurrence of Five Pathogenic *Legionella* spp. And Two Free-Living Amoebae Species in a Complete Drinking Water System and Cooling Towers. Pathogens.

[29] Lu J., Buse H., Struewing I., Zhao A., Lytle D., Ashbolt N. (2017). Annual variations and effects of temperature on *Legionella* spp. And other potential opportunistic pathogens in a bathroom. Environ. Sci. Pollut. Res..

[30] Totaro M., Valentini P., Costa A.L., Frendo L., Cappello A., Casini B., Miccoli M., Privitera G., Baggiani A. (2017). Presence of *Legionella* spp. in Hot Water Networks of Different Italian Residential Buildings: A Three-Year Survey. Int. J. Environ. Res. Public Health.

[31] Hull N.M., Holinger E.P., Ross K.A., Robertson C.E., Harris J.K., Stevens M.J., Pace N.R. (2017). Longitudinal and Source-to-Tap New Orleans, LA, USA. Drinking Water Microbiology. Environ. Sci. Technol..

[32] Li L., Qin T., Li Y., Zhou H., Song H., Ren H., Li L., Li Y., Zhao D. (2015). Prevalence and Molecular Characteristics of Waterborne Pathogen *Legionella* in Industrial Cooling Tower Environments. Int. J. Environ. Res. Public Health.

[33] Zhang L., Li Y., Wang X., Shangguan Z., Zhou H., Wu Y., Wang L., Ren H., Hu Y., Lin M. (2017). High Prevalence and Genetic Polymorphisms of *Legionella* in Natural and Man-Made Aquatic Environments in Wenzhou, China. Int. J. Environ. Res. Public Health.

[34] Nguyen C., Elfland C., Edwards M. (2012). Impact of advanced water conservation features and new copper pipe on rapid chloramine decay and microbial regrowth. Water Res..

[35] Rhoads W.J., Pruden A., Edwards M.A. (2016). Survey of green building water systems reveals elevated water age and water quality concerns. Environ. Sci. Water Res. Technol..

[36] Centers for Disease Control and Prevention *Legionella* (Legionnaires’ Disease and Pontiac Fever). History, Burden, and Trends. https://www.cdc.gov/legionella/about/history.html.

[37] Centers for Disease Control and Prevention Legionnaires’ Disease Surveillance Summary Report, United States 2014–2015. https://www.cdc.gov/legionella/health-depts/surv-reporting/2014-15-surv-report-508.pdf.

[38] Dimitriadi D., Velonakis E. (2014). Detection of *Legionella* spp. From Domestic Water in the Prefecture of Arta 2014, Greece. J. Pathog..

[39] Fleres G., Couto N., Lokate M., van der Sluis L.W.M., Ginevra C., Jarraud S., Deurenberg R.H., Rossen J.W., García-Cobos S., Friedrich A.W. (2018). Detection of *Legionella anisa* in Water from Hospital Dental Chair Units and Molecular Characterization by Whole-Genome Sequencing. Microorganisms.

[40] Mee-Marquet N., van der Domelier A.-S., Arnault L., Bloc D., Laudat P., Hartemann P., Quentin R. (2006). *Legionella anisa*, a Possible Indicator of Water Contamination by *Legionella pneumophila*. J. Clin. Microbiol..

[41] Edagawa A., Kimura A., Miyamoto H. (2019). Investigations on Contamination of Environmental Water Samples by *Legionella* using Real-Time Quantitative PCR Combined with Amoebic Co-Culturing. Biocontrol Sci..

[42] Laganà P., Facciolà A., Palermo R., Delia S. (2019). Environmental Surveillance of Legionellosis within an Italian University Hospital-Results of 15 Years of Analysis. Int. J. Environ. Res. Public Health.

[43] Leoni E., De Luca G., Legnani P.P., Sacchetti R., Stampi S., Zanetti F. (2005). *Legionella* waterline colonization: Detection of *Legionella* species in domestic, hotel and hospital hot water systems. J. Appl. Microbiol..

[44] Fragou K., Kokkinos P., Gogos C., Alamanos Y., Vantarakis A. (2012). Prevalence of *Legionella* spp. In water systems of hospitals and hotels in South Western Greece. Int. J. Environ. Health Res..

[45] Thornley C.N., Harte D.J., Weir R.P., Allen L.J., Knightbridge K.J., Wood P.R.T. (2017). *Legionella longbeachae* detected in an industrial cooling tower linked to a legionellosis outbreak, New Zealand, 2015; possible waterborne transmission?. Epidemiol. Infect..

[46] Smith A.F., Huss A., Dorevitch S., Heijnen L., Arntzen V.H., Davies M., Robert-Du Ry van Beest Holle M., Fujita Y., Verschoor A.M., Raterman B. (2019). Multiple Sources of the Outbreak of Legionnaires’ Disease in Genesee County, Michigan, in 2014 and 2015. Environ. Health Perspect..

[47] Schwake D.O., Garner E., Strom O.R., Pruden A., Edwards M.A. (2016). *Legionella* DNA Markers in Tap Water Coincident with a Spike in Legionnaires’ Disease in Flint, MI. Environ. Sci. Technol. Lett..

[48] Hamilton K.A., Hamilton M.T., Johnson W., Jjemba P., Bukhari Z., LeChevallier M., Haas C.N., Gurian P.L. (2019). Risk-Based Critical Concentrations of *Legionella pneumophila* for Indoor Residential Water Uses. Environ. Sci. Technol..

[49] Legionellosis: Risk Management for Building Water Systems. Standard 188-2018. American Society of Heating, Refrigeration and Air-Conditioning Engineers (ASHRAE). https://www.ashrae.org/technical-resources/bookstore/ansi-ashrae-standard-188-2018-legionellosis-risk-management-for-building-water-systems.

[50] (2016). Recognition, Evaluation and Control of Legionella n Building Water Systems, 2nd ed.; American Industrial Hygiene Association (AIHA): Fairfax, VA, USA. https://online-ams.aiha.org/amsssa/ecssashop.show_product_detail?p_mode=detail&p_product_serno=1047&p_cust_id=257816&p_order_serno=&p_promo_cd=&p_price_cd=&p_category_id=&p_session_serno=5604896&p_trans_ty=.

[51] New York State Department of Health Protection against Legionella. https://www.health.ny.gov/environmental/water/drinking/legionella/.

[52] Schneiders S., Hechard T., Edgren T., Avican K., Fällman M., Fahlgren A., Wang H. (2021). Spatiotemporal Variations in Growth Rate and Virulence Plasmid Copy Number during Yersinia pseudotuberculosis Infection. Infect. Immun..

[53] Katz S.M., Hammel J.M. (1987). The effect of drying, heat, and pH on the survival of *Legionella pneumophila*. Ann. Clin. Lab. Sci..

[54] Nazarian E.J., Bopp D.J., Saylors A., Limberger R.J., Musser K.A. (2008). Design and implementation of a protocol for the detection of *Legionella* in clinical and environmental samples. Diagn. Microbiol. Infect. Dis..

[55] Cross K.E., Mercante J.W., Benitez A.J., Brown E.W., Diaz M.H., Winchell J.M. (2016). Simultaneous detection of *Legionella* species and *L**. anisa*, *L. bozemanii*, *L. longbeachae* and *L. micdadei* using conserved primers and multiple probes in a multiplex real-time PCR assay. Diagn. Microbiol. Infect. Dis..

