# Supplementary material for: Water Age Effects on the Occurrence and Concentration of Legionella Species in the Distribution System, Premise Plumbing, and the Cooling Towers
Source: Microorganisms. 2021 Dec 31;10(1):81. doi: 10.3390/microorganisms10010081 (PMC8778510; doi:10.3390/microorganisms10010081)
Supplement: Supplementary file 1 [file microorganisms-10-00081-s001.zip › microorganisms-1512544-supplementary.pdf]

**SI Table S1.** General and specific *Legionella* primers and probes. 23S pan-*Legionella* is conserved by all species of *Legionella;* but the probes were uniquely designed to specifically identify each species: *L. micdadei, L. anisa, L.bozemanii,* and *L. longbeachae*. All primers and probes were previously designed and validated by the authors listed in the references below.

| Target Species | Primer/Probe name | Primer/Probe Sequence | Amplicon length (bp) | Reference |
|---|---|---|---|---|
| *Legionella species* | 23SF<br>23SR<br>23SP | 5′-CCCATGAAGCCCGTTGAA-3′<br>5′-ACAATCAGCCAATTAGTACGAG TTAGC-3′<br>5′-HEX-TCCACACCTCGCCTATCAACGTCGTAGT-BHQ1-3′ | 92 | [54] |
| *L. pneumophila* | mipF<br>mipR<br>mipP | 5′-AAAGGCATGCAAGACGCTATG-3′<br>5′-GAAACTTGTTAAGAACGTCTTTCATTTG-3′<br>5′-FAM-TGGCGCTCAATTGGCTTTAACCGA-BHQ1-3′ | 78 | [54] |
| *L. micdadei*<br>*L. anisa*<br>*L. bozemanii*<br>*L. longbeachae* | Pan-Legionella F<br>Pan-Legionella R<br>LmicdadeiP<br>LanisaP<br>Lbozemanii<br>LlongbeachaeP | 5′-GTACTAATTGGCTGATTGTCTTG-3′<br>5′-TTCACTTCTGAGTTCGAGATGG-3′<br>5′-FAM-AGCTGATTGGTTAATAGCCCAATCGG-BHQ1-3′<br>5′-HEX-CTCAACCTACGCAGAACTACTTGAGG-BHQ1-3′<br>5′-FAM-TACGCCCATTCATCATGCAAACCAGnT-BHQ1-3′<br>5′-HEX-CTGAGTATCATGCCAATAATGCGCGC-BHQ1-3′ | Not available | [55] |

## 1. ddPCR Information

### *Legionella* genomic DNA

Five *Legionella* species were obtained from American Type Culture Collection (ATCC®) and are described in detail in the experimental section. The positive genomic DNA was extracted using a QIAamp DNA Mini Kit (Qiagen, CA, USA). The positive genomic DNA concentrations were estimated using a UV spectrometry (Nanodrop). Serial dilutions of the stock genomic DNA were eluted using DNAase/RNAase free water and were performed to produce up to five ten-fold dilutions. Each serial dilution volume was 100 µl—this volume was used to reduce the number of freeze-thawing of the originally stock. A temperature gradient (52-65°C) was initially run to determine the optimal degree for primer/probe annealing for amplification of each ten-fold dilution. For each assay, the dilution factor, $10^{-5}$, was the optimal (not saturated) factor for the ddPCR reaction. For each assay, a freshly thawed aliquot genomic DNA was used without further dilution and stored at 4°C until the completion of the ddPCR reaction. Storing at 4°C was ideal in the event the assay needed to be repeated; this procedure allowed the analysts to reduce the freeze-thawing of the DNA. After the successful ddPCR reaction, all genomic DNA were stored at -20°C.

### Sample DNA

The environmental DNA concentrations were estimated using a UV spectrometry (Nanodrop). Environmental DNA was extracted using a crude DNA extraction method and described in detail in the experimental section. All samples were stored at -80°C and analyzed by ddPCR within a month of extraction. For each ddPCR assay, a freshly thawed aliquot (~60 µl) sample was used without any dilution. Afterwards each sample was held in the 4°C refrigerator for up to 48 hours —in the event the assay needed to be re-run; if not, any remaining sample was discarded.

### Assay Validation

Each assay was optimized by running an annealing temperature gradient using a Bio-Rad C1000 Touch thermal cycler. The assay format is a duplex reaction. The details of the experimental set are described in the full body of the manuscript. There was no amplification of either target in buffered solution (phosphate buffered water). Also, there was not any cross-reactivity with either positive control. The detection limit was determined for all assays by multiplying the minimum number of droplets (3) as well as back calculating the initial and final volume of water collected and assayed. As described in the manuscript, the assays lower detection limit detectable by the ddPCR method is 1.3 GC/100 mL. This number is calculated using the filtration volume (10 L), and the concentrated volume (10 mL), which was used for extracting the environmental DNA. Each positive genomic DNA was spiked to determine any inhibition of the assay and no inhibition was observed for either of the duplex assays.

### Data Analysis (Partition classification method)

The analysis software that was used was QuantaSoft v.1.7.4.0917. The threshold was manually set, and it was based off the positive and negative controls. The classification mode was a histogram with two colored channels (channel 1 and channel 2). There were three positive and

negative controls to match triplicate reactions per sample. Because there were replicates of each sampling site, replication of the entire experiment process (including all preanalytical steps such as collection, extraction, and measuring) was evaluated. Repeatability of each technical and biological replicate varied across assays, ranging from 40 to 100%.

**SI Table S2.** Raw data of Five Disease Relevant *Legionella* species

| AlshaeData | | | | | | | |
|---|---|---|---|---|---|---|---|
| Site_ID | Date | Legionella_pneumophila | Legionella_anisa | Legionella_micdadei | Legionella_bozemanii | Legionella_longbeachae | Sample |
| RES_IN_1 | 7/15/2019 | 1.5 | 0 | 0 | 2.1 | 0 | RES |
| RES_IN_2 | 7/23/2019 | 1.3 | 0 | 0 | 0 | 0 | RES |
| RES_IN_3 | 7/29/2019 | 1.6 | 0 | 0 | 1.8 | 0 | RES |
| RES_IN_4 | 8/6/2019 | 1.4 | 0 | 3.3 | 1.3 | 0 | RES |
| RES_IN_5 | 8/13/2019 | 1.2 | 0 | 0 | 0 | 0 | RES |
| RES_IN_6 | 8/20/2019 | 1.5 | 0 | 0 | 1.8 | 0 | RES |
| RES_EF_1 | 7/15/2019 | 1.8 | 0 | 0 | 1.6 | 0 | RES |
| RES_EF_2 | 7/23/2019 | 1.4 | 0 | 0 | 1.3 | 0 | RES |
| RES_EF_3 | 7/29/2019 | 1.7 | 0 | 0 | 1.6 | 0 | RES |
| RES_EF_4 | 8/6/2019 | 1.9 | 0 | 0 | 1.8 | 0 | RES |
| RES_EF_5 | 8/13/2019 | 1.5 | 0 | 0 | 1.6 | 0 | RES |
| RES_EF_6 | 8/20/2019 | 0 | 0 | 0 | 1.3 | 0 | RES |
| FA_IN | 8/12/2019 | 1.5 | 0 | 0 | 0 | 0 | FA |
| FA_1C | 8/12/2019 | 0 | 1.1 | 1.1 | 0 | 1.1 | FA |
| FA_1H | 8/12/2019 | 0 | 1.5 | 0 | 0 | 0 | FA |
| FA_2C | 8/12/2019 | 0 | 0 | 0 | 0 | 1.3 | FA |
| FA_2H | 8/12/2019 | 0 | 2.1 | 0 | 1.8 | 1.8 | FA |
| FA_IN | 9/3/2019 | 1.7 | 0 | 1.5 | 0 | 1.7 | FA |
| FA_1C | 9/3/2019 | 1.5 | 0 | 0 | 0 | 1.1 | FA |
| FA_1H | 9/3/2019 | 1.9 | 0 | 0 | 0 | 1.7 | FA |
| FA_2C | 9/3/2019 | 0 | 0 | 0 | 0 | 0 | FA |
| FA_2H | 9/3/2019 | 0 | 1.5 | 0 | 0 | 0 | FA |
| FA_IN | 9/16/2019 | 0 | 0 | 1.7 | 0 | 0 | FA |
| FA_1C | 9/16/2019 | 1.4 | 0 | 0 | 0 | 0 | FA |
| FA_1H | 9/16/2019 | 1.4 | 1.4 | 0 | 0 | 1.3 | FA |
| FA_2C | 9/16/2019 | 0 | 0 | 0 | 1.4 | 1.4 | FA |
| FA_2H | 9/16/2019 | 0 | 0 | 0 | 0 | 0 | FA |
| ERC_IN | 8/19/2019 | 1.4 | 0 | 1.7 | 1.2 | 1.1 | ERC |
| ERC_1C | 8/19/2019 | 1.8 | 0 | 0 | 0 | 0 | ERC |
| ERC_1H | 8/19/2019 | 0 | 0 | 1.4 | 0 | 0 | ERC |
| ERC_IN | 9/9/2019 | 0 | 0 | 0 | 1.8 | 1.3 | ERC |
| ERC_1C | 9/9/2019 | 1.5 | 0 | 0 | 0 | 0 | ERC |
| ERC_1H | 9/9/2019 | 0 | 0 | 0 | 0 | 0 | ERC |
| ERC_IN | 9/23/2019 | 0 | 0 | 1.5 | 1.4 | 1.2 | ERC |
| ERC_1C | 9/23/2019 | 1.1 | 0 | 0 | 0 | 0 | ERC |
| ERC_1H | 9/23/2019 | 0 | 0 | 3.6 | 1.8 | 1.7 | ERC |
| CT1 | 7/25/2019 | 2.7 | 2.1 | 0 | 4.3 | 0 | CT |
| CT4 | 7/31/2019 | 3.7 | 2.7 | 3.2 | 3.1 | 0 | CT |
| CT7 | 8/7/2019 | 2.6 | 2.5 | 1.7 | 2.6 | 1.7 | CT |
| CT8 | 8/14/2019 | 2.1 | 0 | 0 | 1.6 | 1.4 | CT |
| CT10 | 8/21/2019 | 3.2 | 1.2 | 0 | 2.3 | 0 | CT |
| CT11 | 8/21/2019 | 0 | 0 | 0 | 4.5 | 1.7 | CT |
